# The Role of Point-of-Care C-Reactive Protein Testing in Antibiotic Prescribing for Respiratory Tract Infections: A Survey among Swiss General Practitioners

**DOI:** 10.3390/antibiotics11050543

**Published:** 2022-04-19

**Authors:** Nahara Anani Martínez-González, Andreas Plate, Levy Jäger, Oliver Senn, Stefan Neuner-Jehle

**Affiliations:** 1 Institute of Primary Care, University of Zurich and University Hospital of Zurich, Pestalozzistrasse 24, CH-8091 Zurich, Switzerland; andreas.plate@usz.ch (A.P.); levy.jaeger@usz.ch (L.J.); oliver.senn@usz.ch (O.S.); stefan.neuner-jehle@usz.ch (S.N.-J.); 2 Department of Health Sciences and Medicine, University of Lucerne, Frohburgstrasse 3, CH-6002 Lucerne, Switzerland

**Keywords:** survey, antibiotic prescribing, appropriate prescribing, antibiotic resistance, respiratory tract infections, point-of-care test, c-reactive protein, primary care, general practice, decision-making, knowledge, awareness, attitudes, barriers, facilitators

## Abstract

Understanding the decision-making strategies of general practitioners (GPs) could help reduce suboptimal antibiotic prescribing. Respiratory tract infections (RTIs) are the most common reason for inappropriate antibiotic prescribing in primary care, a key driver of antibiotic resistance (ABR). We conducted a nationwide prospective web-based survey to explore: (1) The role of C-reactive protein (CRP) point-of-care testing (POCT) on antibiotic prescribing decision-making for RTIs using case vignettes; and (2) the knowledge, attitudes and barriers/facilitators of antibiotic prescribing using deductive analysis. Most GPs (92–98%) selected CRP-POCT alone or combined with other diagnostics. GPs would use lower CRP cut-offs to guide prescribing for (more) severe RTIs than for uncomplicated RTIs. Intermediate CRP ranges were significantly wider for uncomplicated than for (more) severe RTIs (*p* = 0.001). Amoxicillin/clavulanic acid was the most frequently recommended antibiotic across all RTI case scenarios (65–87%). Faced with intermediate CRP results, GPs preferred 3–5-day follow-up to delayed prescribing or other clinical approaches. Patient pressure, diagnostic uncertainty, fear of complications and lack of ABR understanding were the most GP-reported barriers to appropriate antibiotic prescribing. Stewardship interventions considering CRP-POCT and the barriers and facilitators to appropriate prescribing could guide antibiotic prescribing decisions at the point of care.

## 1. Introduction

Antibiotic resistance (ABR) is an established threat to public health globally. The inappropriate use of antibiotics to treat infectious diseases in humans is a key driver of the rising rates of ABR [1]. As much as 50% of the antibiotics prescribed by general practitioners (GPs) are considered inappropriate, posing a significant challenge to primary healthcare and making GPs essential players in reducing suboptimal prescribing [2,3,4]. It is crucial to understand the factors that guide GPs’ decision-making in antibiotic prescribing in order to develop stewardship strategies to change GPs’ prescribing behaviours and curb ABR [5,6].

Diagnostic testing has a critical role in guiding healthcare decision-making as it reduces diagnostic uncertainty and contributes to rational prescribing [1,7]. As an additional diagnostic procedure, C-reactive protein (CRP) point-of-care testing (POCT) can reduce immediate antibiotic prescribing for respiratory tract infections (RTIs), the most common indications for antibiotic prescriptions in primary care [5,8]. Furthermore, international clinical guidelines have facilitated the evidence-based use of CRP-POCT. Guidance includes recommendations on the use of specific CRP cut-offs to withhold (e.g., CRP < 20 mg/L or <50 mg/L) or prescribe (CRP ≥ 100 mg/L) antibiotics for RTIs [9,10,11,12]. Clinicians’ decision-making remains challenging when CRP results are intermediate (ranges between the cut-offs, e.g., 20–99 mg/L) [13,14]. In such cases, clinical guidelines often recommend delayed prescribing [13,14].

Contrary to many European countries [15], POCT is widely implemented and used in Switzerland [16]. However, little is known about GPs’ use of POCT for disease management, especially how GPs utilise CRP results to inform their prescribing decisions for RTIs [16,17,18,19]. Research from psychological theories shows that clinicians’ knowledge, attitudes and beliefs about the disease, ABR and the consequences of prescribing decisions influence this process [20,21,22]. Factors such as patients’ expectations, patient–physician communication, time pressure and underestimating ABR also increase the likelihood of inappropriate antibiotic prescribing [21,23]. We set out to explore Swiss GPs’ decision-making patterns focusing on the role of CRP in guiding antibiotic prescribing for RTIs. In addition, we aimed to explore GPs’ knowledge and attitudes toward antibiotic prescribing and ABR, including their perceived barriers and facilitators to (appropriate) antibiotic prescribing.

## 2. Materials and Methods

We conducted a prospective web-based survey that follows the Checklist for the reporting of Results of Internet E-Surveys (CHERRIES) reporting guidelines (see Supplementary Materials) [24].

### 2.1. Participants

We used a purposive sampling strategy to invite GPs practising in Switzerland’s German- and French-speaking regions to participate in our survey. Invitations in flyer format were inserted in the print Journal Primary and Hospital Care [25,26]. The journal has a print circulation of 7000 copies. The flyer provided a brief description of the study with an incentive to participate, donating ten Swiss francs (~11 USD) to a charity project. It also included the web link and the quick response code to access the questionnaire in German and French. We ran the survey for fourteen weeks. The Association of Swiss General Practitioners and Paediatricians (Hausärzte Schweiz—mfe) sent a reminder at week ten as part of their regular electronic newsletter.

### 2.2. Survey Instrument and Data Collection

We implemented a structured self-administered web-based questionnaire and hosted it on the SurveyMonkey platform (SurveyMonkey Inc., San Mateo, CA, USA). We developed the questionnaire by consulting the literature on evidence from similar topics of antibiotic prescribing [20,21]. A group of five practising GPs, including members from our institute and external GPs, pilot-tested the questionnaire and checked the appropriateness of technical content, language, comprehensibility and time. We adapted the final version of the questionnaire and limited survey entries to one Internet protocol address per participant. We mainly collected quantitative data to address our research questions and included a qualitative data section from open-ended questions. Most of the questions required answers in tick-box format.

### 2.3. Clinical Case-Vignettes

We integrated four disease-specific case vignettes that mimicked real-life clinical scenarios with varying disease severity and patient-related factors for two main types of RTIs (see Supplementary Materials). As upper RTIs vignettes, we included a generally healthy patient with a cough and an elderly patient with comorbidities and sore throat. As lower RTIs vignettes, we included chronic obstructive pulmonary disease (COPD) in two forms: a patient with COPD in stable condition with cough (uncomplicated COPD) and a patient with exacerbated COPD. We use the term RTIs throughout to refer to this set of RTIs.

### 2.4. Survey Structure

We structured the survey by grouping questions around five issues in five sections (see Supplementary Materials File S1):

Section one explored the decision-making practices by using diagnostics for the management of clinical cases. All GPs were asked to indicate if they would perform a diagnostic procedure or proceed differently (prescribe directly, no prescribing and no diagnostics, or a different strategy) as an initial approach to managing the cases. The section is focused on GPs answering “yes” to the use of diagnostics. GPs were prompted to select the POCTs of their choice from a list.

Section two investigated the decision-making practices in antibiotic prescribing for all clinical cases when using CRP among GPs choosing POCTs from section one. GPs were asked to provide the cut-offs to which they refer as a guide to prescribe (above cut-off) or withhold (below cut-off) antibiotics, assuming that a CRP-POCT was the only available result. GPs could select the cut-offs from a range of numbers (0–150) displayed on a sliding bar, or they could type the numbers in a box. GPs also had to select (from a list) the antibiotics they would prescribe if CRP-POCT results were above their prescribing cut-off and the approach for further management if CRP-POCT showed results between the cut-offs (i.e., intermediate ranges). The list of antibiotics comprised a set of those most prescribed by Swiss GPs according to the FIRE database project (Family Medicine ICPC-Research using Electronic Medical Records) [27].

Sections three to five applied to all GPs answering the survey. Section three prompted them to indicate whether they relied on evidence-based guidance when answering sections one and two. Section four explored aspects of knowledge and attitudes on ABR and antibiotic prescribing using four statements in a 5-item Likert type format. Section five explored the barriers and facilitators to appropriate antibiotic prescribing, asking participants to provide as free text the factors perceived as important barriers (max. 3) and facilitators (max. 3). In a final section, we sought GPs’ demographic and professional details, including whether GPs worked in dispensing practices, i.e., where GPs themselves supply the medication to their patients. Physician drug dispensing is partly allowed in Switzerland, and rules to ban or allow such dispensing are determined at the cantonal (“regional”) level [28].

### 2.5. Statistical and Content Analyses

We exported data from the SurveyMonkey platform as comma-separated files and processed and analysed data using Microsoft Office Excel and the statistical software R 4.0.3 (R Foundation for Statistical Computing, Vienna, Austria), including the Likert and boxplot (graphics) packages [29,30]. We used descriptive statistics to compute the number of events and corresponding proportions (*n* and %), means and standard deviations (SD) or medians and interquartile ranges (IQR). Using the Wilson method, we computed the 95% confidence intervals (CIs) of proportions for the selected POCTs and antibiotics, and for the strategies GPs would follow if faced with CRP intermediate ranges [31]. We performed Wilcoxon rank-sum tests to assess the differences in gender among participants’ characteristics and the differences in the distribution of CRP intermediate ranges between the uncomplicated and (more) severe clinical cases and considered *p* < 0.05 statistically significant. We performed a deductive content analysis of the factors perceived as barriers and facilitators using methods similar to those reported by Björkman et al. [32]. Two analysts developed a broad set of categories following a pre-defined list of themes based on the literature [20,21]. The two analysts became familiar with the data separately, classifying the factors into a broad category set, comparing the findings, resolving differences by discussion and determining a broad set of categories. One analyst iteratively developed a sublayer of categories to code the themes further, which was reviewed by a third author familiar with content analysis and the questionnaire. All authors approved the final tabulated categories and (sub)themes frequency.

## 3. Results

### 3.1. Characteristics of Respondents

**Figure 1:**
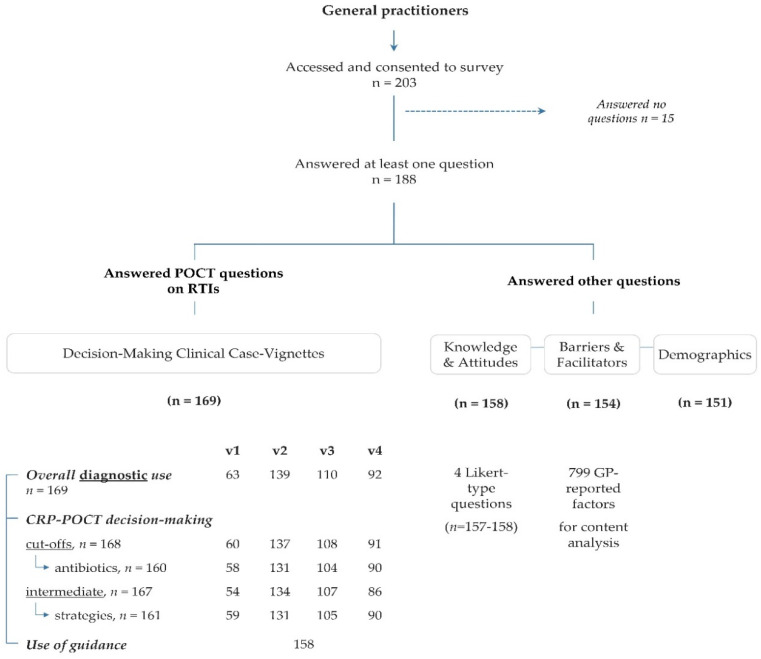
Flow chart of participants in a survey exploring point-of-care-testing (POCT) decision-making for respiratory tract infections (RTIs) and the behavioural factors influencing antibiotic prescribing. 169 GPs would perform POCT for the initial management of RTIs; 19 GPs would proceed differently (Supplementary Materials). GPs provided the C-reactive protein (CRP) cut-offs guiding their prescribing decisions, chose the antibiotics they would prescribe and selected the strategies they would follow if faced with CRP intermediate values. Case vignettes: healthy patient with a cough (v1), uncomplicated chronic obstructive pulmonary disease (COPD) (v2), exacerbated COPD (v3) and an elderly patient with comorbidities and sore throat (v4).

A total of 203 GPs accessed and consented to participate in the survey, of whom 188 (92.6%) answered at least one question (Figure 1). Between 151 (74.4%) and 169 (83.3%) of GPs accessing and consenting to participate answered the different survey sections. Among 151 GPs completing their demographics, the mean age was 52 years (SD 10.0), and 77 (51.0%) were male (Table 1). On average, GPs would see 22 (SD 7.0) patients per day and had 22 years (SD 10.8) of professional experience. Compared to male GPs, female GPs had significantly fewer years of professional experience (*p* = 0.001), lower employment rates (*p* < 0.001) and provided significantly fewer consultations (*p* = 0.003). Most GPs worked in group, network-affiliated and physician dispensing practices.

### 3.2. Use and Choice of Diagnostics to Guide Disease Management

**Figure 2:**
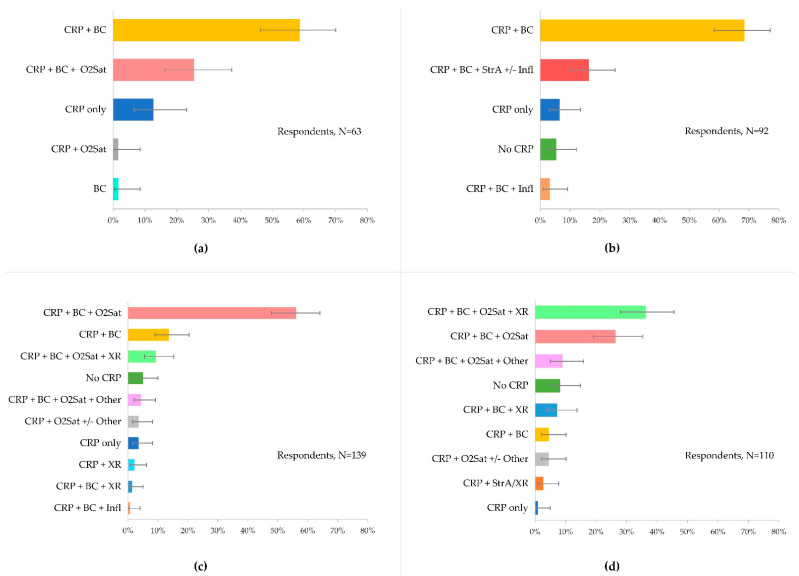
Diagnostic procedures that GPs would select to support clinical decision-making for further disease management. Error bars denote 95% confidence intervals. (**a**) Healthy patient with cough, (**b**) Elderly with comorbidities and sore throat, (**c**) Uncomplicated COPD, and (**d**) Exacerbated COPD. CRP, C-reactive protein; BC, blood count; O2Sat, oxygen saturation; No CRP, other POCTs excluding CRP; Infl, swab for rapid influenza test; StrA, swab for group A streptococci; XR, chest X-ray; Other, include PCR virus, swab for multiplex PCR and swab for culture within others. COPD, chronic obstructive pulmonary disease.

A total of 19 of the 188 GPs would follow approaches other than performing diagnostics for the initial management of RTIs (see Supplementary Materials). Figure 2 shows the diagnostic procedures that 169 GPs would select to manage the different RTI scenarios before prescribing. The proportion of respondents opting for diagnostic testing varied by clinical scenario and ranged between 37.3% (*n* = 63) and 82.3% (*n* = 139). CRP-POCT alone or in combination with other diagnostics was the most preferred choice. Across all clinical scenarios, at least 92% (range: 92–98%) of the GPs selected CRP-POCT. CRP plus blood count (BC) was the diagnostic combination most preferred by at least 58.7% (*n* = 37/63; 95% CIs 46.4 to 70.0) and up to 68.5% (*n* = 63/92; 95% CIs 58.4 to 77.1) of the GPs in the healthy and the elderly cases, respectively. CRP combined with BC and oxygen saturation (O2Sat) was most preferred by 56.1% (*n* = 78/139; 95% CIs 47.8 to 64.1) of GPs to manage uncomplicated COPD. CRP plus BC, O2Sat and chest radiography were most preferred by 36.4% (*n* = 40/110; 95% CIs 28.0 to 45.7) GPs to manage exacerbated COPD.

### 3.3. Use of CRP-POCT for Respiratory Tract Infections

#### 3.3.1. CRP to Guide Antibiotic Prescribing

**Figure 3:**
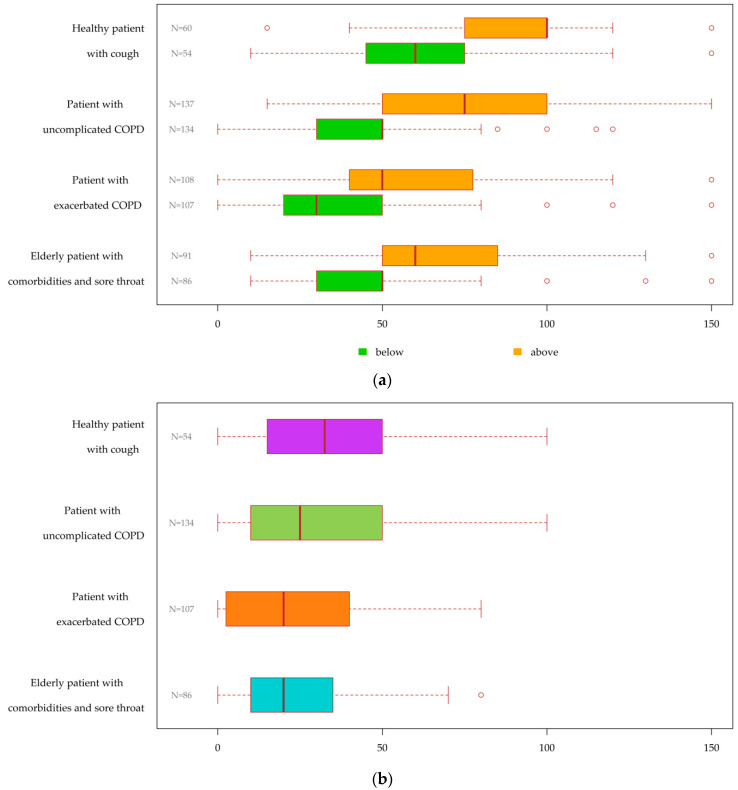
Distribution of CRP-POCT cut-offs that would guide GPs’ antibiotic prescribing decision-making, assuming that CRP is the only test result available: (**a**) CRP cut-offs (mg/L): below CRP cut-offs, i.e., GPs’ guide for withholding antibiotics and above CRP cut-offs, i.e., GPs’ guide for prescribing antibiotics; (**b**) Intermediate CRP ranges, i.e., values between the above and below CRP cut-offs. CRP, C-reactive protein; POCT, point of care test; COPD, chronic obstructive pulmonary disease.

Figure 3a illustrates the distribution of CRP-POCT cut-offs reported by GPs who would perform further diagnostics for each clinical scenario. Assuming that CRP was the only result available, GPs would use lower CRP cut-offs to guide antibiotic prescribing for exacerbated COPD and the elderly with sore throat cases than for the healthy with cough and uncomplicated COPD cases. Figure 3b illustrates the distribution of CRP intermediate ranges available from 167 (99.4%) of the GPs reporting (above and below) CRP-POCT cut-offs. Analysis of intermediate CRP ranges by RTI severity, i.e., uncomplicated (healthy cough + uncomplicated COPD) versus more severe (exacerbated + elderly sore throat) RTIs, showed significantly wider intermediate ranges for the uncomplicated than for the (more) severe RTIs (*p* = 0.001) (Supplementary Materials).

#### 3.3.2. CRP Cut-Offs and Antibiotics

**Figure 4:**
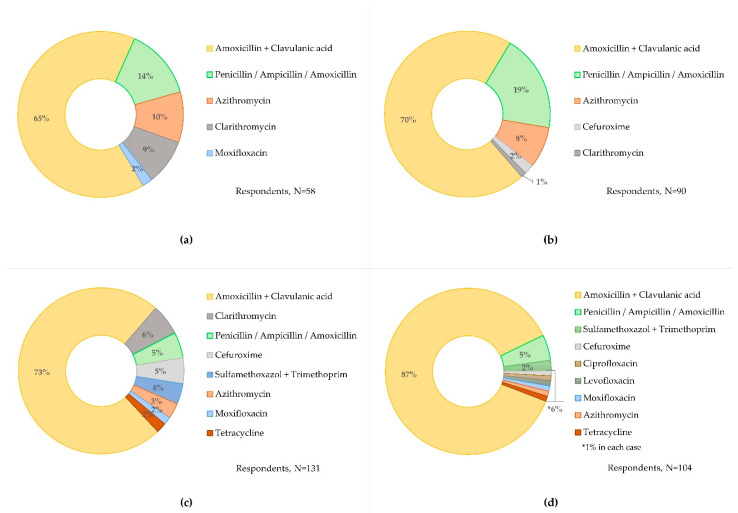
Antibiotics selected when GPs faced CRP-POCT prescribing cut-offs, assuming that CRP is the only test result available. (**a**) Healthy patient with cough, (**b**) Elderly with comorbidities and sore throat, (**c**) Uncomplicated COPD, and (**d**) Exacerbated COPD. CRP, C-reactive protein; POCT, point of care test, COPD, chronic obstructive pulmonary disease.

Figure 4 shows the choice of antibiotics that 160 (95.2%) GPs would prescribe across the four clinical scenarios when faced with CRP-POCT prescribing cut-offs. Amoxicillin plus clavulanic acid was the preferred antibiotic across all clinical scenarios, accounting for at least 65.5% (healthy case, *n* = 38/58; 95% CIs 52.7 to 76.4) and up to 86.5% (exacerbated COPD case, *n* = 90/104; 95% CIs 78.7 to 91.8) of the GP responses. Four of the five antibiotics preferred to treat the healthy with cough and the elderly with sore throat cases were the same in both scenarios. Similarly, nine of the ten antibiotics preferred to treat the uncomplicated and exacerbated COPD cases were the same in both scenarios.

#### 3.3.3. CRP Intermediate Ranges and Disease Management

**Figure 5:**
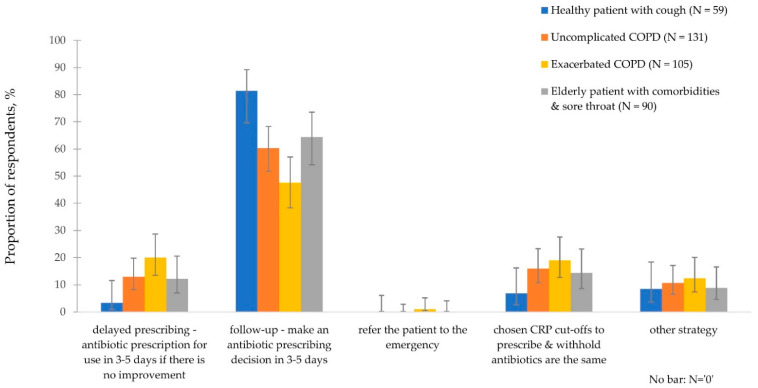
Approaches selected for further management of RTIs when GPs faced CRP-POCT intermediate results. Error bars denote 95% confidence intervals.

Figure 5 shows the approach that 161 (96.4%) GPs would follow for further disease management of each clinical case when faced with CRP-POCT intermediate ranges. Across all clinical scenarios, GPs would most commonly follow-up patients and make an antibiotic prescribing decision in 3–5 days. While this was most frequent for the healthy with cough case, it was the least common approach for the exacerbated COPD case, accounting for 81.4% (*n* = 48/59; 95% CIs 69.6 to 89.3) and 47.6% (*n* = 50/105; 95% CIs 38.3 to 57.1) of the responses, respectively. Second, GPs would apply delayed prescribing, i.e., issuing an antibiotic prescription for use only if there was no improvement in 3–5 days, most commonly for exacerbated COPD and least frequently for the healthy with cough case, with 20.0% (*n* = 21/105; 95% CIs 13.5 to 28.6) and 3.4% (*n* = 2/59; 95% CIs 0.9 to 11.5) respectively. Between 8.5% (*n* = 5/59; 95% CIs 3.7 to 18.4) and 12.4% (*n* = 13/105; 95% CIs 7.4 to 20.0) of the GPs would perform a different (‘other’) strategy such as seeing the patient as soon as the next day or performing further diagnostics (Supplementary Materials).

### 3.4. Use of Evidence-Based Guidance for Decision-Making

Among 158 (93.5%) GPs responding to sections one and two, 14.7% (*n* = 23/158) used evidence-based information or tools for guiding their decision, e.g., local (COPD/pneumonia) guidelines, Centor/McIsaac score, or GOLD directives (Supplementary Materials).

### 3.5. Knowledge, Awareness and Attitudes of Antibiotic Prescribing and Antibiotic Resistance

**Figure 6:**
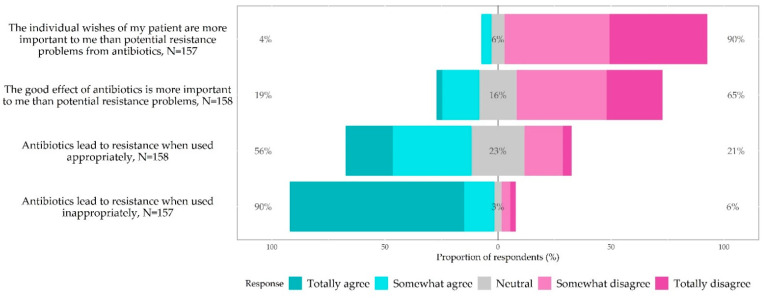
Relative frequencies of responses to questions on knowledge and attitudes toward antibiotic prescribing and antibiotic resistance. Question: Based on your experience as a doctor, to what extent do you agree with the following statements?

Figure 6 illustrates the relative frequency of responses from 158 GPs to knowledge, awareness and attitudes questions about ABR and antibiotic prescribing. The answers revealed that 89.8% (*n* = 141/157) of the GPs disagreed that their patients’ wishes were more important than potential ABR problems. Similarly, 64.6% (*n* = 102/158) disagreed that the good effect of antibiotics was more important than potential ABR problems, although 16.4% (*n* = 26/158) neither agreed nor disagreed with this statement. While 90.4% (*n* = 142/157) of GPs agreed that antibiotics lead to ABR if used inappropriately, 55.7% (*n* = 88/158) also thought antibiotics lead to resistance if used appropriately.

### 3.6. Barriers and Facilitators to Appropriate Antibiotic Prescribing

A total of 154 GPs reported 843 factors that they perceived as barriers or facilitators to appropriate antibiotic prescribing. We excluded forty-four entries as they were unclear phrases. The analysis of content retrieved six thematical categories with the classification of 799 factors: 44.3% (*n* = 354) barriers and 55.7% (*n* = 445) facilitators (Table 2). Overall, 87.1% (*n* = 696/799) of the factors were classified as patient-, physician- or clinical-related themes. The most common barriers were patient demand or expectation of receiving antibiotics (71.2%, *n* = 79/111), fear of complications or treatment side effects (34.5%, *n* = 40/116), diagnostic uncertainty (40.5%, *n* = 47/116) and lack of knowledge/understanding of antibiotics or ABR (32.8%, *n* = 38/116). The most common facilitators were informed patient (30.3%, *n* = 10/33), physicians’ experience (31.0%, *n* = 27/87), good access/availability to laboratory/POCT (27.5%, *n* = 64/233) and clear/accurate clinical diagnosis (18.5%, *n* = 43/233). Themes on regulation measures, society-related topics and evidence-based sources accounted for up to 11.0% of all the factors.

## 4. Discussion

To the best of our knowledge, this is the first survey in the Swiss GP setting which explores the role of CRP-POCT in guiding antibiotic prescribing decision-making for RTIs and the behavioural factors influencing antibiotic prescribing. The more complex the clinical cases were, the more POCTs GPs selected, mostly CRP-POCT alone or combined with other diagnostics. GPs preferred 3–5-day follow-up visits to delayed prescribing if faced with intermediate CRP-POCT values. The main barriers to appropriate antibiotic prescribing were patients’ wishes and expectations, followed by GP-related factors such as knowledge about ABR, fear of disease-related complications and diagnostic uncertainty.

Among all POCTs in our study, CRP is highly preferred alone or combined with other diagnostics to support clinical decision-making. GPs tend to request CRP and BC the most, followed by CRP combined with O2Sat and/or chest X-rays. Only <10% of the GPs would request diagnostics other than CRP.

Consistent with these results, CRP-POCT is one of the two most common GP-requested POCTs in Swiss primary care, generally performed along with BC and potentially overused with substantial variation between GPs [33]. In patients with RTIs, POCT is requested for 42–69% of all cases [16], and CRP (≤69%) and BC (≤62%) are the most performed [16,18,19]. Higher POCT use, including CRP, has been associated with significant reductions (21–37%) in antibiotic prescriptions [17]. Moreover, POCTs’ use appears highest in German-speaking regions and lowest in Italian- and French-speaking regions [17].

Our results are also similar to those reported in Dutch and Scandinavian countries. A recent study on POCT for RTIs across eighteen European countries found that CRP and/or StrA are performed in >65% of consultations in Denmark, Norway and the Netherlands, and BC is often performed in Norway, Ukraine, Greece, Croatia and Moldova [34]. Interestingly, antibiotic prescribing was associated with patient-related factors and country but not with POCT use. Other Scandinavian studies also found a potential CRP overuse with CRP-POCT performance rates of 31–66% for RTI consultations [35,36,37,38]. Yet, antibiotic consumption in the Scandinavian and Dutch communities is lower than in many European countries [39].

GPs relied on the clinical presentation rather than clinical guidance and based their interpretation of CRP thresholds on patients’ characteristics and clinical severity. They selected lower CRP cut-offs to guide prescribing for (more) severe RTIs (e.g., exacerbated COPD) and higher CRP cut-offs for uncomplicated RTIs (e.g., healthy individuals with cough). Similarly, Swiss GPs in one study guided their decisions using CRP-POCT as part of the clinical examination, a symptoms assessment and BC [18]. In other two Swiss studies, GPs used CRP thresholds of 50 mg/L as a proxy for disease severity and prescribing decisions [16,18]. However, typical viral RTIs were also frequently (51%) treated with antibiotics in one study [16].

GPs in our study tended to choose broad-spectrum antibiotics (BSA). Faced with an antibiotic prescribing decision, most GPs selected amoxicillin/clavulanic in each clinical scenario, most often for COPD cases. Local [40] and international [41,42] guidance agrees that in ambulatory patients with exacerbated COPD, antibiotics may be initiated if specific criteria for symptoms severity and CRP > 40 mg/L are met. If prescribing, amoxicillin/clavulanic acid is recommended as a first-line treatment for acute COPD exacerbations but not for uncomplicated COPD [40,41,42]. Most international guidelines also agree that routine antibiotics should be avoided for patients with upper acute RTIs who are not systematically unwell or at high risk of complications [9,10,12]. A critical drawback to the overuse of BSA is their high potential for increasing the risk of resistant infections if used when not needed [43]. The Swiss national surveillance for antibiotic resistance (ANRESIS) in the outpatient setting [44] and an observational study in Swiss general practice [45] also identified this BSA overuse.

Intermediate CRP ranges were significantly wider for uncomplicated than for (more) severe RTIs. Randomised controlled trials (RCTs) and non-randomised studies have shown that (wider) intermediate CRP levels (e.g., 20–99 mg/L) could lead to major clinical uncertainty and higher antibiotic prescribing due to the challenge in interpreting the risk of deterioration [46,47,48]. Research has also shown that communicating intermediate CRP results to patients can be challenging [14], and with intermediate results, patients tend to re-consult more often [49]. RCTs have shown that communication skills adjunct to CRP-POCT can safely reduce unnecessary antibiotic prescribing [50]. Accordingly, international clinical guidelines also suggest that delayed prescribing can be helpful in decisions surrounding intermediate CRP levels when illness severity does not require immediate antibiotics [10,12,42].

GPs would most commonly prefer 3–5-day follow-up to delayed prescribing when faced with intermediate CRP results. Research shows that doctors prefer patients to re-consult due to the need for self-, and/or patient-, reassurance about disease progression, due to feeling uncomfortable in passing over clinical responsibility to the patient, or due to a preference to avoid conflict [51]. Re-consultations, however, may often be unnecessary and can increase workload and the risk of unnecessary antibiotic prescribing [51]. A meta-analysis has shown that delayed prescribing is a safe and effective alternative to immediate antibiotic prescribing [52]. It reduces re-consultation rates without increasing the risk of complications while increasing patient satisfaction. It also leads to reduction in the belief in antibiotics and in antibiotic use [53]. In addition, clear patient treatment advice can be a crucial adjunct to delayed prescribing, enhancing these effects [53,54,55,56].

GPs generally had a sound awareness of antibiotics and ABR, but it appeared that some GPs were not very familiar with the ABR concept. ABR develops naturally with antibiotics, but inappropriate antibiotic use accelerates such a process [1]. Research shows that knowledge, attitudes and beliefs about the disease and the consequences of their prescribing decisions can influence physicians’ decision-making [20,21,22,57,58].

Several of the barriers and facilitators increasingly acknowledged as affecting prescribing decisions [20,21,22,23] have also been identified in our study. GPs perceived patient-, physician- and clinical-related themes as major barriers and facilitators to appropriate antibiotic prescribing. Our findings that the demand for, or the expectation of receiving, antibiotics accounted for 71% of the patient-related barriers suggest that GPs perceive pressure from patients. Research shows that patients’ expectations can drive GPs to maintain a good patient–physician relationship, influencing antibiotic prescribing and sometimes overriding evidence-based recommendations [23,59,60,61]. GPs also recognised diagnostic uncertainty and the (associated) fear of complications as frequent barriers to appropriate antibiotic prescribing. The influence of diagnostic uncertainty on prescribing behaviour is still not well understood. Nevertheless, it has been associated with inappropriate antibiotic prescribing, especially in primary and emergency care [62,63,64,65] and with unplanned hospitalisations [66].

Lack of knowledge/understanding of antibiotics or ABR and clinical resources (e.g., restricted consultation time and workload) were common barriers to appropriate prescribing for our GPs, as described in European studies [67,68]. Limited time for consultations and an increased number of patients per day, for example, are linked to limited patient–physician communication and to reducing physicians’ ability to provide advice efficiently, leading to irrational antibiotic prescribing [69]. Although cited the least, lack of clear and timely updated clinical guidance was perceived as a barrier. The use of guidelines can be hindered at the same time by insufficient consultation time, the evidence and evidence-based skills, as well as GPs’ attitudes and patient-related factors [70,71]. Research also shows, however, that prescribing practices can be modified when physicians’ knowledge, beliefs, attitudes and skills are shaped around ABR [71].

The majority of GP-cited facilitators were clinical- and GP-related. The former include structural factors such as diagnostics’ availability in GPs’ practice and clear clinical diagnosis, while the latter cover experience and training aspects. The use of diagnostics has been linked to reduction in uncertainty, enhancement of GPs’ confidence in prescribing decisions and managing patients’ expectations, and improvement in antibiotic prescribing behaviour [14,72]. Several studies have shown that interventions including CRP-POCT and CRP guidance, educational aspects on POCTs and antibiotic use, and communication skills training can significantly reduce antibiotic prescribing in primary care [50,73,74,75,76,77,78]. These findings have been backed up by a relatively recent meta-analysis [8].

Clinical guidance accounted for only a minority of the facilitators and only a minority of GPs stated that they used evidence-based information to guide their survey answers. Future implementation of guidance should incorporate strategies to overcome the low level of engagement in using evidence-based information.

### 4.1. Strengths and Limitations

This study is the first to explore Swiss GPs’ decision-making process for RTIs and the behaviour-related factors driving antibiotic prescribing decisions. Using case vignettes allowed a multifaceted exploration of the complex real-life aspects of performing POCT, particularly CRP-POCT, and the clinical reasoning behind diagnostic and prescribing decisions. It was thus possible to gain insights on the factors on which further research could focus to understand antibiotic prescribing decision-making better. The use of POCT, particularly CRP-POCT, for the clinical management of RTIs is relevant to stakeholders and policymakers at a national level. The role that currently available diagnostics could play in strengthening antibiotic stewardship needs to be considered. National efforts have readily acknowledged POCT as a key solution to the irrational use of antibiotics [79,80]. The WHO’s objectives also highlight the need for effective diagnostics to improve antibiotic prescribing [1].

We could not cover the wide range of RTIs as it would have required an extensive survey and a lengthy participation time. The small set of RTIs included in our survey are highly relevant for GPs, since the set includes those most prescribed with antibiotics in general practice [44,81]. Judgement about the appropriateness of antibiotic prescribing was beyond the scope of our study. We could not estimate the response rate since we did not have access to the mailing lists of the journal and the mfe association which were used to send the survey invitations. These lists include active GPs, retired physicians or GPs who no longer work in routine care. Thus, although we know the number of GPs accessing the questionnaire, we could not determine the exact number of GPs who received and noticed the invitation but decided to ignore it. Unfortunately, the COVID-19 pandemic highly impacted the GP workforce by substantially challenging GPs’ workload, likely preventing them from answering the survey. As a survey of purposive sampling, our sample was not selected randomly, but it was based on existing mailing lists. Thus, the study’s representativeness is limited and prone to self-selection bias. We acknowledge that the factors influencing prescribing decision-making may vary across the different language-speaking regions, which we could not investigate due to the small sample size. Since all answers were self-reported, they are prone to recall and desirability bias, as it may be anticipated from web-based surveys [82].

### 4.2. Policy Considerations and Future Research

The insights from this study represent a window of opportunity for policymaking and future research. There are no specific guidelines on the use of POCTs. Addressing the appropriate use of POCTs could be a cornerstone of best-practice guidance for managing infectious diseases. Local COPD guidelines have integrated CRP cut-offs prescribing guidance [40], but a CRP-guided algorithm has yet to be considered in other key guidelines [83,84,85]. GP efforts to prioritise interventions for better healthcare have so far included the recommendation to not use CRP or BC as sole routine interventions in viral infections [86]. Although the relationship between antibiotic use and POCT is not necessarily causal [34], countries with implemented POCT have shown lower antibiotic consumption than countries where POCT is not widely implemented. Scandinavian studies have also shown that a lack of proper guideline-based CRP-POCT can lead to CRP overuse and antibiotic overprescribing due to poor interpretation of CRP levels, especially in upper RTIs [38,48]. In the Netherlands, which has consistently shown one of the lowest antibiotic prescribing rates in Europe [87], CRP-POCT implementation accompanied indications for CRP and guided interpretation of CRP cut-offs [11]. Other international initiatives have endorsed CRP-POCT and delayed prescribing to assist clinical assessment and decision-making [9,10,88].

Given the wide implementation and daily use of CRP-POCT in routine care, Switzerland could naturally stand at the forefront of the efforts to reduce sub-optimal prescribing and reduce ABR. It could provide insights into the (real-life) application of CRP-POCT guidance, especially on approaches to deal with intermediate CRP ranges. As supported by meta-analyses, a clinical algorithm including CRP-POCT and CRP cut-off guidance with indications for CRP and delayed prescribing could strengthen CRP’s utility, supporting decision-making, especially where there is uncertainty about antibiotic prescribing [8,52]. It could reduce inappropriate antibiotic prescribing and re-consultations. CRP-POCT has also been linked to less antibiotic prescribing for RTIs in nursing homes [78] and COPD exacerbations in primary care clinics [89,90]. Diagnostic reasoning and training in communication skills could provide GPs with the necessary elements to gain (more) confidence and inform POCT results with (more) reassurance [91,92,93].

Research shows that multifaceted stewardship interventions could more likely reduce (suboptimal) antibiotic prescribing while increasing the use of recommended antibiotics [94,95]. Considering physicians’ and patients’ barriers and facilitators toward antibiotic prescribing, interventions could encompass several elements such as CRP-POCT-guided algorithm, GP communication skills training, diagnostic reasoning *and* patient education strategies.

## 5. Conclusions

Given the extensive use of CRP-POCT for decision-making in RTIs by Swiss GPs in our study, guidelines and policies to help improve treatment decisions may benefit from integrating CRP-guided antibiotic prescribing recommendations. Similarly, these results could be considered in countries where CRP-POCT is widely used. Since delayed prescribing appears to be an uncommon practice so far, it could be a promising strategy for intermediate CRP-POCT results. Additionally, our findings on knowledge, attitudes and barriers and facilitators towards antibiotic prescribing could inform the design of future antibiotic stewardship interventions.

## Figures and Tables

**Table 1:** Demographic and professional characteristics of study participants.

Participants Characteristics		*p*-Value
Responders with demographic data, *n*	151	
Gender, *n* (%)		
Female	74 (49.0)	
Male	77 (51.0)	
Age in years, mean (SD)		0.001
Overall	52 (10.1)	
Female	49 (9.0)	
Male	54 (10.5)	
Years of professional experience, mean (SD)		0.001
Overall	22 (10.8)	
Female	19 (9.7)	
Male	25 (11)	
Percentage of employment, % mean (SD)		<0.001
Overall	74 (23.5)	
Female	66 (19.4)	
Male	81 (25.0)	
Number of patients per day, mean (SD)		0.003
Overall	22 (7.0)	
Female	19 (9.7)	
Male	25 (11.0)	
Type of practice, *n* (%)		
Individual practice	34 (22.5)	
Dual practice	30 (19.9)	
Group practice	80 (53.0)	
Hospital outpatient consultation	7 (4.6)	
Network affiliation practice, *n* (%)		
Network affiliated practice	109 (72.2)	
Non-network affiliated practice	38 (25.2)	
Hospital outpatient consultation	4 (2.6)	
Dispensing type practice, *n* (%)		
Self-dispensing practice	92 (60.9)	
Non-self-dispensing practice	56 (37.1)	
Hospital outpatient consultation	3 (2.0)	

**Table 2:** Factors most frequently cited as barriers and facilitators of appropriate antibiotic prescribing.

Theme/Subtheme		Factors, *n* (%)
BARRIERS *	**Total**	**354 (44)**
	Patient-related	111
	patient wish, request, demand, pressure or expectation to receive antibiotics	79 (71.2)
	negative or defensive patient attitude, rejection of antibiotics by patient	8 (7.2)
	GP-related	116
	fear of complications or side effects of treatment	40 (34.5)
	lack of knowledge, awareness, consciousness or understanding of antibiotics and judicious antibiotic use, prescribing and ABR	38 (32.8)
	Clinical-related	116
	diagnostic uncertainty, uncertain or unclear clinical picture	47 (40.5)
	clinical or practice resources: consultation time too short or time pressure	16 (13.8)
	clinical or practice resources: follow-up consultations under time pressure or not possible	11 (9.5)
	Regulation measures/sources	8
	lack of guidance or clear recommendations for treatment and management of disease, or guidance not available in a timely fashion (immediately, when it is needed)	3 (37.5)
	lack of effective or stricter measures or procedures to appropriately moderate antibiotics use	2 (25.0)
	Society-related	2
	environmental impact	1 (50.0)
	pressure from others or the society to be fit	1 (50.0)
	Evidence-based	1
	need for better, new or updated evidence-based medical resources and information	1 (100)
**FACILITATORS ^‡^**	**Total**	**445 (56)**
	Patient-related	33
	(well) informed patient or (good) patient information e.g., leaflets, websites, etc	10 (30.3)
	patient wish, request or expectation to be treated without antibiotics or according to the evidence	5 (15.2)
	patient consent or patient collaboration or patient cooperation	5 (15.2)
	GP-related	87
	physicians’ experience	27 (31.0)
	(more) education (prevention) and (good) training for GPs about judicious antibiotic consumption, prescribing and ABR, e.g., leaflets and programs	13 (14.9)
	knowledge, awareness or perception of disease, e.g., risks, effects, treatment choice, complications (e.g., hospitalisation), effects of broad-spectrum antibiotics	8 (9.2)
	Clinical-related	233
	good access to or availability of (additional, specific or appropriate choice of) diagnostic tests or laboratory in the practice setting	64 (27.5)
	clear or accurate clinical picture, diagnosis or course of disease	43 (18.5)
	clear symptomatology, underlying condition, severity of disease or comorbidities	32 (13.7)
	Regulation measures/sources	76
	clear, effective, properly updated or rapidly accessible local and international guidelines for disease management and routine prescribing procedures	56 (73.7)
	clear, effective or properly updated guidance specific for antibiotic prescribing and use	8 (10.5)
	Society-related	5
	Media or media reports “available” to the whole population and society	2 (40.0)
	(increasing or growing) population knowledge or understanding of antibiotics and ABR	1 (20.0)
	Evidence-based	11
	use of new or updated evidence-based resources, information, science and research	10 (90.9)

Note: Other categories within each subtheme adding to 100% are not included in this table; * What are three main factors you perceive as barriers to prescribing antibiotics appropriately? **^‡^** What are three main factors you perceive as facilitators to prescribing antibiotics appropriately?

## Data Availability

The data presented in this study are available upon request from the corresponding author after approval of the internal review board.

## Supplementary Materials

The following are available online at https://www.mdpi.com/article/10.3390/antibiotics11050543/s1, Table S1. Checklist for Reporting Results of Internet E-Surveys (CHERRIES). Table S2. Clinical case vignettes. Table S3. Other approaches selected as initial management for the clinical cases. Table S4. CRP cut-offs used as a guide for withholding and prescribing antibiotics, assuming CRP is the only test result available. Table S5. Intermediate CRP ranges based on CRP cut-offs. Table S6. Strategies for disease management when CRP concentrations are intermediate. File S1. Questionnaire.
