# Supplementary material for: The Role of Point-of-Care C-Reactive Protein Testing in Antibiotic Prescribing for Respiratory Tract Infections: A Survey among Swiss General Practitioners
Source: Antibiotics (Basel). 2022 Apr 19;11(5):543. doi: 10.3390/antibiotics11050543 (PMC9137646; doi:10.3390/antibiotics11050543)
Supplement: Supplementary file 1 [file antibiotics-11-00543-s001.zip › antibiotics-1660599-supplementary.pdf]

Table S1. Checklist for Reporting Results of Internet E-Surveys (CHERRIES).

Item Category	*Checklist Item*	*Explanation*	*Page Number*
Design	Describe survey design	Describe target population, sample frame. Is the sample a convenience sample? (In "open" surveys this is most likely.)	4, Methods section
IRB (Institutional Review Board) approval and informed consent process	IRB approval	Mention whether the study has been approved by an IRB.	6, Methods section, Page 16
	Informed consent	Describe the informed consent process. Where were the participants told the length of time of the survey, which data were stored and where and for how long, who the investigator was, and the purpose of the study?	4, Methods section, Page 16
	Data protection	If any personal information was collected or stored, describe what mechanisms were used to protect unauthorized access.	Guaranteed with user name and password of the responsible researchers' login to the SurveyMonkey account
Development and pre-testing	Development and testing	State how the survey was developed, including whether the usability and technical functionality of the electronic questionnaire had been tested before fielding the questionnaire.	4, Methods section
Recruitment process and description of the sample having access to the questionnaire	Open survey versus closed survey	An "open survey" is a survey open for each visitor of a site, while a closed survey is only open to a sample which the investigator knows (password-protected survey).	4, Methods section: Open survey
	Contact mode	Indicate whether or not the initial contact with the potential participants was made on the Internet. (Investigators may also send out questionnaires by mail and allow for Web-based data entry.)	4, Methods section: E-mail and post
	Advertising the survey	How/where was the survey announced or advertised? Some examples are offline media (newspapers), or online (mailing lists – If yes, which ones?) or banner ads (Where were these banner ads posted and what did they look like?). It is important to know the wording of the announcement as it will heavily influence who chooses to participate. Ideally the survey announcement should be published as an appendix.	4, Methods section: Invitations in flyer format inserted in the issue of a print journal
Survey administration	Web/E-mail	State the type of e-survey (eg, one posted on a Web site, or one sent out through e-mail). If it is an e-mail survey, were the responses entered manually into a database, or was there an automatic method for capturing responses?	4, Methods section: web-based survey
	Context	Describe the Web site (for mailing list/newsgroup) in which the survey was posted. What is the Web site about, who is visiting it, what are visitors normally looking for? Discuss to what degree the content of the Web site could pre-select the sample or influence the results. For example, a survey about vaccination on a anti-	4, Methods section: with a neutral web survey appearance questionnaire developed using the commercial web tool SurveyMonkey Inc., San Mateo, California, USA

		immunization Web site will have different results from a Web survey conducted on a government Web site	
	Mandatory/voluntary	Was it a mandatory survey to be filled in by every visitor who wanted to enter the Web site, or was it a voluntary survey?	4, Methods section: voluntary
	Incentives	Were any incentives offered (eg, monetary, prizes, or non-monetary incentives such as an offer to provide the survey results)?	4, Methods section: offered to donate ten Swiss francs to a charity project for every participant completing the questionnaire
	Time/Date	In what timeframe were the data collected?	4, Methods section: 14 weeks
	Randomization of items or questionnaires	To prevent biases items can be randomized or alternated.	4, Methods section: No
	Adaptive questioning	Use adaptive questioning (certain items, or only conditionally displayed based on responses to other items) to reduce number and complexity of the questions.	Methods and Results section: yes
	Number of Items	What was the number of questionnaire items per page? The number of items is an important factor for the completion rate.	51 (maximum)
	Number of screens (pages)	Over how many pages was the questionnaire distributed? The number of items is an important factor for the completion rate.	22 (maximum)
	Completeness check	It is technically possible to do consistency or completeness checks before the questionnaire is submitted. Was this done, and if "yes", how (usually JAVAScript)? An alternative is to check for completeness after the questionnaire has been submitted (and highlight mandatory items). If this has been done, it should be reported. All items should provide a non-response option such as "not applicable" or "rather not say", and selection of one response option should be enforced.	Answers to all questions were voluntary, and we included no completeness checks during the survey.
	Review step	State whether respondents were able to review and change their answers (eg, through a Back button or a Review step which displays a summary of the responses and asks the respondents if they are correct).	"Go back" and "proceed/next" buttons were used so that participants could switch between pages and change their answers.
Response rates	Unique site visitor	If you provide view rates or participation rates, you need to define how you determined a unique visitor. There are different techniques available, based on IP addresses or cookies or both.	Only participants completing at least the first page were identified as respondents; calculation of view and participation rate was therefore not possible. Also declared in the Discussion / Limitations of the study.
	View rate (Ratio of unique survey visitors/unique site visitors)	Requires counting unique visitors to the first page of the survey, divided by the number of unique site visitors (not page views!). It is not unusual to have view rates of less than 0.1 % if the survey is voluntary.	
	Participation rate (Ratio of unique	Count the unique number of people who filled in the first survey page (or agreed to participate, for example by checking a checkbox), divided by visitors who visit	

	visitors who agreed to participate/unique first survey page visitors)	the first page of the survey (or the informed consents page, if present). This can also be called “recruitment” rate.	
	Completion rate (Ratio of users who finished the survey/users who agreed to participate)	The number of people submitting the last questionnaire page, divided by the number of people who agreed to participate (or submitted the first survey page). This is only relevant if there is a separate “informed consent” page or if the survey goes over several pages. This is a measure for attrition. Note that “completion” can involve leaving questionnaire items blank. This is not a measure for how completely questionnaires were filled in. (If you need a measure for this, use the word “completeness rate”.)	Results section: Overall, 203 GPs accessed and consented to participate in the survey. Of those, 188 (93%) completed at least one question. Between 151 (74.4%) and 169 (83.3%) of GPs accessing and consenting to participate in the survey completed the different survey sections.
Preventing multiple entries from the same individual	Cookies used	Indicate whether cookies were used to assign a unique user identifier to each client computer. If so, mention the page on which the cookie was set and read, and how long the cookie was valid. Were duplicate entries avoided by preventing users access to the survey twice; or were duplicate database entries having the same user ID eliminated before analysis? In the latter case, which entries were kept for analysis (eg, the first entry or the most recent)?	Methods section: cookies to assign unique identifiers to each client device and thus prevent double answers, limited survey entries to one internet protocol address per participant.
	IP check	Indicate whether the IP address of the client computer was used to identify potential duplicate entries from the same user. If so, mention the period of time for which no two entries from the same IP address were allowed (eg, 24 hours). Were duplicate entries avoided by preventing users with the same IP address access to the survey twice; or were duplicate database entries having the same IP address within a given period of time eliminated before analysis? If the latter, which entries were kept for analysis (eg, the first entry or the most recent)?	
	Log file analysis	Indicate whether other techniques to analyze the log file for identification of multiple entries were used. If so, please describe.	None.
	Registration	In “closed” (non-open) surveys, users need to login first and it is easier to prevent duplicate entries from the same user. Describe how this was done. For example, was the survey never displayed a second time once the user had filled it in, or was the username stored together with the survey results and later eliminated? If the latter, which entries were kept for analysis (eg, the first entry or the most recent)?	No login was required.
Analysis	Handling of incomplete questionnaires	Were only completed questionnaires analyzed? Were questionnaires which terminated early (where, for example, users did not go through all questionnaire pages) also analyzed?	All questionnaires with answers to at least one question were analysed.

	Questionnaires submitted with an atypical timestamp	Some investigators may measure the time people needed to fill in a questionnaire and exclude questionnaires that were submitted too soon. Specify the timeframe that was used as a cut-off point, and describe how this point was determined.	Time was recorded and no atypical timestamps were detected.
	Statistical correction	Indicate whether any methods such as weighting of items or propensity scores have been used to adjust for the non-representative sample; if so, please describe the methods.	No statistical correction was implemented

This checklist has been modified from Eysenbach G. Improving the quality of Web surveys: the Checklist for Reporting Results of Internet E-Surveys (CHERRIES). J Med Internet Res. 2004 Sep 29;6(3):e34 [erratum in J Med Internet Res. 2012; 14(1): e8.]. Article available at https://www.jmir.org/2004/3/e34/; erratum available https://www.jmir.org/2012/1/e8/. Copyright ©Gunther Eysenbach. Originally published in the Journal of Medical Internet Research, 29.9.2004 and 04.01.2012.
This is an open-access article distributed under the terms of the Creative Commons Attribution License (https://creativecommons.org/licenses/by/2.0/), which permits unrestricted use, distribution, and reproduction in any medium, provided the original work, first published in the Journal of Medical Internet Research, is properly cited.

Table S2. Clinical case vignettes.

**Case vignette 1:** Healthy patient with cough	**Case vignette 2:** Patient with uncomplicated COPD	**Case vignette 3:** Patient exacerbated COPD	**Case vignette 4:** Elderly patient with various comorbidities and sore throat
For this case vignette, assume that the following occurs in your practice.<br>Patient information: female, 53 years old.<br>Presenting complaint: non-productive cough for 3 days. General feeling of sickness with arthralgia and myalgia. Occasional headache. Fever not measured. No dyspnoea.<br>Past medical history: uneventful.<br>Medication: None.<br>Social/family/travel/allergy history: uneventful.<br>Vital parameters: temperature 38.1°C; BP 118/76 mmHg; Pulse 91 / min; RR 14/min.<br>Physical: good general condition. Posterior pharyngeal wall not reddened. Tonsils normal, no exudation. No cervical lymphadenopathy. Pulmonary auscultation without findings.	For this case vignette, assume that the following occurs in your practice.<br>Patient information: female, 53 years old.<br>Presenting complaint: increasing cough for 3 days. General feeling of sickness with arthralgia and myalgia. Occasional headache. Fever not measured. Known dyspnoea on great exertion, currently no increase. No increase in the amount of sputum, sputum not purulent.<br>Past medical history: COPD GOLD II, group B, stable. Last FEV1 at 66% of predicted.<br>Medication: Tiotropium bromide 1x18 µg, salbutamol for 2 days without improvement.<br>Social/family/travel/allergy history: uneventful.<br>Vital parameters: temperature 38.1 °C; BP 138/76 mmHg; Pulse 91/min; RR 14/min.<br>Physical: good general condition. Intraoral redness, no exudation. No cervical lymphadenopathy. Discretely obstructive sounds on pulmonary auscultation.	For this case vignette, assume that the following occurs in your practice.<br>Patient information: female, 53 years old.<br>Presenting complaint: increasingly productive cough with purulent discharge for 5 days. Increasing dyspnoea on exertion. General feeling of sickness<br>with arthralgia and myalgia. Occasional headache. Fever not measured.<br>Past medical history: COPD GOLD II, group B, last exacerbation 3 months ago. Last FEV1 at 66% of predicted.<br>Medication: Tiotropium bromide 1x18 µg, salbutamol for 2 days without improvement.<br>Social/family/travel/allergy history: uneventful.<br>Physical: temperature 38.1 °C; BP 138/76 mmHg; Pulse 91/min; RR 14/min.<br>Status: Significantly reduced general condition. No intraoral redness, no exudation. No cervical lymphadenopathy. Obstructive sounds on pulmonary auscultation.	For this case vignette, assume that the following occurs in your practice.<br>Patient information: female, 73 years old.<br>Presenting complaint: sore throat for 3 days. General feeling of sickness with arthralgia and myalgia. No headache. Fever not measured. No cough.<br>Past medical history: coronary and hypertensive heart disease, type 2 diabetes, and hypercholesterolemia. Left-sided breast cancer 10 years ago with mastectomy (free from recurrence).<br>Medication: acetylsalicylic acid 100 mg, perindopril/indapamide 10/2.5, metformin, rosuvastatin.<br>Social/family/travel/allergy history: uneventful.<br>Vital parameters: temperature: 38.1 °C; BP: 138/76 mmHg; Pulse: 91/min; RR: 14/min.<br>Physical: Good general condition for age. Posterior pharyngeal wall reddened. Tonsils normal, no exudation. No cervical lymphadenopathy. Pulmonary auscultation without findings.

Abbreviations: BP, blood pressure; COPD, chronic obstructive pulmonary disease; FEV1, forced expiratory volume in 1 second; GOLD, Global Initiative for Chronic Obstructive Lung Disease; RR, respiratory rate.

Table S3. Other approaches selected as initial management for the clinical cases.

Other approaches	Healthy patient with cough	Patient with uncomplicated COPD	Patient with exacerbated COPD	Elderly patient with comorbidities & sore throat
**no diagnostics and no antibiotics**	18	6	0	13
**immediate prescribing**	0	5	11	0
**another strategy, no POCT**	1	4	3	1
**answered further diagnostics but had missing POCT data**	0	1	0	0
**no answer**	0	3	5	5
**Total**	19	19	19	19

Note. COPD, Chronic Obstructive Pulmonary Disease.

Table S4. CRP cut-offs used as a guide for withholding and prescribing antibiotics, assuming CRP is the only test result available.

Case-Vignette / CRP data (mg/L)	Healthy patient with cough		Patient with uncomplicated COPD		Patient with exacerbated COPD		Elderly patient with comorbidities & sore throat	
	withhold	prescribe	withhold	prescribe	withhold	prescribe	withhold	prescribe
**Respondents, N (%)**	54 (90)	60 (100)	134 (97.8)	137 (100)	107 (99.1)	108 (100)	86 (94.5)	91 (100)
**range (min-max)**	10-150	15-150	0-120	15-150	0-150	0-150	10-150	10-150
**median**	60	100	50	75	30	50	50	60
**IQR (Q1-Q3)**	46.3-75	75-100	30-50	50-100	20-50	40-76.25	30-50	50-85

Note. CRP, C-reactive protein test; IQR, Interquartile Ranges; Withhold = what would be a CRP cut-off (in mg / L) below which you would not prescribe an antibiotic in this case?; Prescribe = what would be a CRP cut-off (in mg / L) above which you would prescribe an antibiotic in this case?

Table S5. Intermediate CRP ranges based on CRP cut-offs.

Case-Vignette / CRP data	Healthy patient with cough	Patient with COPD in normal general condition & cough	Patient with exacerbated COPD	Elderly patient with comorbidities & sore throat
**Respondents, N (%)**	54 (90)	134 (97.8)	107 (99.1)	86 (94.5)
**range (min-max)**	0-100	0-100	0-80	0-80
**median**	32.5	25	20	20
**IQR (Q1-Q3)**	15-50	11.3-48.75	2.5-40	10-35

Note. CRP, C-reactive protein test; IQR, Interquartile Ranges; CRP intermediate ranges for the healthy and uncomplicated COPD were wider than ranges for the exacerbated COPD and elderly patients: $p = 0.001$.

Table S6. Strategies for disease management when CRP concentrations are intermediate.

**Healthy patient with cough**	**COPD in normal general condition with cough**	**Exacerbated COPD**	**Elderly patient with comorbidities and sore throat**
Check-up in two days, in 36 to 48 hours either telephone or appointment in the practice, if the symptoms persist, wait and telephone discussion after 48 hours, symptomatic therapy and information, to be in touch immediately in the event of deterioration and within three days if there is no improvement, control in 1-2 days.	Check-up tomorrow, check sputum; Follow-up check on the following day, check-up after three days, phototherapeutic & control 2-3 days, order in 2 days, my decision would depend very much on how previous infections went with this patient. Check-up, the next day, try with prednisolone, check-up two days, possibly give steroids, a follow-up check-in 1-2 days, 1st or 2nd depending on the patient and day of the week, calling if the patient worsens, I would call the patient to follow-up and check for two days and only then decide whether to prescribe AB, thorax X-ray.	Check-up tomorrow; the patient needs an antibiotic anyway. I would still determine H3 and CRP, follow-up the next day, follow-up in two days. Antibiotic prescription in reserve. Control the next day and then antibiotics and prednisolone; the patient should contact me by phone the next day, steroid inhalation or orally, follow-up check after 1-2 days, earlier check, check the following day with the laboratory, should telephone if worse, otherwise check in three days, I would prescribe the patient an antibiotic for immediate use; I only use the laboratory values to assess the progress, CRP is not a limitation criterion in my opinion.	Check tomorrow, phone inquiries the following day. Clinical follow-up check after two days, thorax X-ray, follow-up check after two days in the laboratory, I would arrange a follow-up check with a decision in 1-2 days, thorax X-ray, thorax X-ray.

Note. Refer to Figure 5 in the manuscript. CRP, C-reactive protein test.

File S1. Questionnaire.

For a detailed description of each case vignette*, please refer to Table S2. Questions listed in Sections I-III were presented for each case vignette, and each case vignette was displayed before the questions.

Section I

1. **Initial approach to decision-making.** Questions for all *participating* GPs who accessed the questionnaire and consented to participate.

   Assume that you have the following patient* in your practice:

Case vignette 1:<br>Healthy patient with cough	Case vignette 2:<br>Patient with uncomplicated COPD	Case vignette 3:<br>Patient with exacerbated COPD	Case vignette 4:<br>Elderly patient with various comorbidities and sore throat

   How would you go about it? – *In that case I would...* (tick box question)

   - …prescribe an antibiotic directly without further diagnostics.
   - ...not initiate any further diagnostics, and I would not prescribe any antibiotics.
   - ...arrange for further diagnostics in order to then make a decision regarding the antibiotic prescription.
   - ...choose a different strategy.

2. **Use of Point of Care Test(-ing) (POCT)**. Questions for GPs selecting option three from above, i.e., GPs who would perform further diagnostics (POCTs) in order to then make a decision regarding the antibiotic prescription.

   What diagnostics would you perform? (multiple answers possible) (tick box question)

   - chest x-ray
   - swab for multiplex PCR (respiratory viruses)
   - swab for multiplex PCR (respiratory bacteria)
   - smear for culture
   - swab for influenza rapid test
   - blood count
   - C-reactive protein (CRP)
   - rapid group a streptococcal test swab
   - oxygen saturation
   - other (please specify)

Section II

1. Decision-making using C-reactive protein (CRP) POCT. Questions for GPs selecting *CRP* as a *diagnostic* procedure for managing cases.

Assume that a CRP value is the only test result available at the moment.

a. How high would be the CRP cut-off (in mg/L) above which you would prescribe an antibiotic in this case? <u>(slider question)</u>

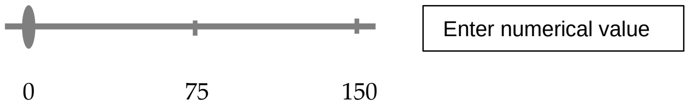

b. What would be a CRP cut-off (in mg/L) below which you would not prescribe an antibiotic in this case? <u>(slider question)</u>

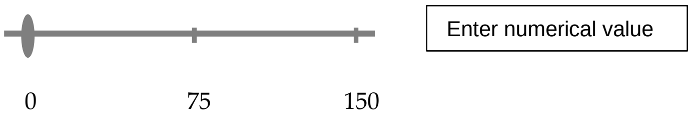

*Answers for a. and b. structured using a numerical slider scale bar with values ranging from 0 to 150. GPs could also enter a value in a text box to reflect the number on the sliding bar.

c. Assume that the CRP value is above the cut-off you specified from which you would prescribe antibiotics. *Which antibiotic would you most likely prescribe?* <u>(tick box question)</u>

- penicillin / ampicillin / amoxicillin
- amoxicillin + clavulanic acid
- cefuroxime
- ciprofloxacin
- levofloxacin
- moxifloxacin
- clarithromycin
- azithromycin
- tetracyclines
- sulfamethoxazole + trimethoprim
- clindamycin

d. Assume that a CRP value is somewhere between the two cut-offs you mentioned. *What approach would you then follow?* <u>(tick box question)</u>

- I would give the patient an antibiotic or an antibiotic prescription that can be used within 3-5 days if there is no improvement. (delayed prescribing)
- I would do a follow-up in 3-5 days and only then make an antibiotic prescribing decision. (short follow-up)
- I would refer the patient to an emergency department.
- Not applicable because the cut-offs chosen to prescribe and withhold antibiotics are the same.
- Others (please specify).

Section III

1. **Use of evidence-based information (decision support) to guide survey answers.** Question for GPs answering the POCTs questions (Sections I and II).

   When answering these questions, I relied on decision-making aids (e.g. guidelines, scores, specialist articles). (tick box and free text box question)

   - Yes
   - No
   - What decision aids did you use?_____________

Section IV

1. **Knowledge and attitudes towards antibiotic prescribing.** Questions for all participating GPs who accessed the questionnaire and consented to participate.

   Based on your experience as a doctor, *to what extent do you agree with the following statements?*

Statement	Totally agree	Somewhat agree	Neutral	Somewhat disagree	Totally disagree
The individual wishes of my patient are more important to me than potential resistance problems from antibiotics					
The good effect of antibiotics is more important to me than potential resistance problems.					
Antibiotics lead to resistance when used appropriately.					
Antibiotics lead to resistance when used inappropriately.					

Section V

1. **Barriers and Facilitators toward antibiotic prescribing.** Questions for all participating GPs who accessed the questionnaire and consented to participate.

   Given your professional experience <u>(free text box questions)</u>…

   a. What are the three most important factors that you perceive as barriers to prescribing antibiotics appropriately

   b. What are the three most important factors that you perceive as facilitators to prescribing antibiotics appropriately?

GPs' demographic and professional information.

Finally, we ask you to provide your demographic information. (tick and text boxes questions)

- How old are you (in years)? ____________________
- What is your gender?
  - ☐ Female ☐ Male ☐ Other
- What is the postal code of your place of work? ____________________
- What is your work experience (in years) as a doctor? ____________________
- What type of practice do you work in?
  - ☐ Single ☐ Double practice ☐ Group practice ☐ Hospital consultation hour
- Is your practice affiliated with a network?
  - ☐ Yes ☐ No ☐ Not applicable
- What is your level of employment? ____________________
- Is there self-dispensation in your practice?
  - ☐ Yes ☐ No ☐ Not applicable
- Approximately how many patients do you see on an average working day? ____________________
